# Supplementary material for: Exploring perceptions of self-stigma of substance use and current alcohol and marijuana use patterns among college students
Source: PLoS One. 2024 Apr 5;19(4):e0301535. doi: 10.1371/journal.pone.0301535 (PMC10997109; doi:10.1371/journal.pone.0301535)
Supplement: S1 Appendix — (DOCX) [file pone.0301535.s001.docx]

**S1 Appendix**

**EFA Results of the Self-Stigma of Substance Use Disorder Scale**

**Statistical Analysis and Results**

To examine the internal structure of the 10-item Stigma of Substance Use Disorder Scale, an exploratory factor analysis (EFA) was conducted using an ML estimator in M*plus* 8.3 (Muthén & Muthén, 1998-2019) on the entire sample of students that were administered the measure (*n =* 2,243). We tested a 1- to 6-factor model utilizing an oblique quartimin rotation. To select the best fitting model, we considered eigenvalues (i.e., Kaiser Rule >1), model fit indices, and interpretability of the identified factors. To evaluate overall model fit, we used model fit criteria suggested by Marsh et al. (2004) including the Comparative Fit Index (CFI) >.90 (acceptable) > .95 (optimal), Tucker-Lewis Index (TLI) >.90 (acceptable) > .95 (optimal), Root Mean Square Error of Approximation (RMSEA) < .06.).

Bartlett’s (1954) test of sphericity was statistically significant (p < .001), suggesting that the data were appropriate for factor analysis. Further, the Kaiser-Meyer-Olkin (Kaiser, 1974) measure of sampling adequacy (.866) indicated that the sample size was appropriate for factor analysis. Results (see <https://doi.org/10.17605/OSF.IO/H8B59>) suggested that a 2-factor solution fit the data best. Specifically, the Kaiser rule suggested a 2-factor solution and model fit was good for a 2-factor solution ([χ^2^(26)=406.23, *p*<.001], CFI = .964, TLI = .938, RMSEA = .081 [90%CIs = 0.074, .088], SRMR = .023). Results from the two-factor model are presented in the table below. Factor 1, negative self-efficacy (NSEF), consists of 5 items that assess perceptions of negative self-efficacy (e.g., “I would feel inadequate if I had a substance use disorder.”). Factor 2, negative self-esteem, consists of 5 items that assess perceptions of negative self-esteem (e.g., “I would feel okay about myself if I had a substance use disorder”; reverse-coded). Internal consistency coefficients for the scores on both factors indicated strong reliability: self-esteem (α = .88) and self-efficacy (α = .84).

**Item Loadings across Two Factors**

| Stigma of Substance Use Disorder Scale Item | Factors | |
| --- | --- | --- |
|  | Self-Efficacy | Self-Esteem |
| I would feel inadequate if I had a substance use disorder. | **.754** | .019 |
| Having a substance use disorder would make me feel less intelligent. | **.763** | -.019 |
| It would make me feel inferior to have a substance use disorder. | **.726** | -.093 |
| If I had a substance use disorder, I would be less satisfied with myself. | **.796** | .034 |
| I would feel worse about myself if I had a substance use disorder. | **.794** | .053 |
| My self-confidence would not be threatened if I had a substance use disorder. (R) | -.135 | **.573** |
| My self-esteem would increase if I had a substance use disorder. (R) | -.049 | **.693** |
| My view of myself would not change just because I had a substance use disorder. (R) | .011 | **.764** |
| I would feel okay about myself if I had a substance use disorder. (R) | .024 | **.818** |
| My self-confidence would remain the same if I had a substance use disorder. (R) | .059 | **.798** |

*Note*. R = reverse coded items.
