# Supplementary material for: Exploring perceptions of self-stigma of substance use and current alcohol and marijuana use patterns among college students
Source: PLoS One. 2024 Apr 5;19(4):e0301535. doi: 10.1371/journal.pone.0301535 (PMC10997109; doi:10.1371/journal.pone.0301535)
Supplement: Attachment — Submitted filename: Response to Reviewers.docx [file pone.0301535.s002.docx]

**Dr. Boshra Ismael Ahmed Arnout,**

**We would like to thank you for the quick and thorough review of our manuscript and for the selection of well-qualified reviewers. We have addressed the reviewer’s concerns and summarized our changes below. By addressing the general journal requirements and reviewers’ comments, we believe our manuscript has been strengthened and will make more of a contribution to the field.**

**Journal (s)' Comments:**

1. **Please ensure that your manuscript meets PLOS ONE's style requirements, including those for file naming. The PLOS ONE style templates can be found at
   https://journals.plos.org/plosone/s/file?id=wjVg/PLOSOne_formatting_sample_main_body.pdf and** 
   [**https://journals.plos.org/plosone/s/file?id=ba62/PLOSOne_formatting_sample_title_authors_affiliations.pdf**](https://journals.plos.org/plosone/s/file?id=ba62/PLOSOne_formatting_sample_title_authors_affiliations.pdf)

*** The manuscript was revised to fit PLOS ONE style requirements per the links provided above and saved as “Revised Manuscript with Track Changes” and “Manuscript” for the track changes and clean versions of the document, respectively.*

2. **Please provide additional details regarding participant consent. In the ethics statement in the Methods and online submission information, please ensure that you have specified (1) whether consent was informed and (2) what type you obtained (for instance, written or verbal, and if verbal, how it was documented and witnessed). If your study included minors, state whether you obtained consent from parents or guardians. If the need for consent was waived by the ethics committee, please include this information. If you are reporting a retrospective study of medical records or archived samples, please ensure that you have discussed whether all data were fully anonymized before you accessed them and/or whether the IRB or ethics committee waived the requirement for informed consent. If patients provided informed written consent to have data from their medical records used in research, please include this information.**

*** The manuscript was revised to highlight additional detail on the informed consent systems employed for this study.*

3. **We note that the grant information you provided in the ‘Funding Information’ and ‘Financial Disclosure’ sections do not match. When you resubmit, please ensure that you provide the correct grant numbers for the awards you received for your study in the ‘Funding Information’ section.**

*** The funding information has been reviewed for consistency and entered in the portal accordingly.*

4. **In your Data Availability statement, you have not specified where the minimal data set underlying the results described in your manuscript can be found.**

**PLOS defines a study's minimal data set as the underlying data used to reach the conclusions drawn in the manuscript and any additional data required to replicate the reported study findings in their entirety. All PLOS journals require that the minimal data set be made fully available. For more information about our data policy, please see** [**http://journals.plos.org/plosone/s/data-availability**](http://journals.plos.org/plosone/s/data-availability)**. Upon re-submitting your revised manuscript, please upload your study’s minimal underlying data set as either Supporting Information files or to a stable, public repository and include the relevant URLs, DOIs, or accession numbers within your revised cover letter. For a list of acceptable repositories, please see http://journals.plos.org/plosone/s/data-availability#loc-recommended-repositories. Any potentially identifying patient information must be fully anonymized.**

**Important: If there are ethical or legal restrictions to sharing your data publicly, please explain these restrictions in detail. Please see our guidelines for more information on what we consider unacceptable restrictions to publicly sharing data: http://journals.plos.org/plosone/s/data-availability#loc-unacceptable-data-access-restrictions. Note that it is not acceptable for the authors to be the sole named individuals responsible for ensuring data access.**

**We will update your Data Availability statement to reflect the information you provide in your cover letter.**

*** The data availability statement has been added to the cover letter.*

5. **Please include your full ethics statement in the ‘Methods’ section of your manuscript file.**

**In your statement, please include the full name of the IRB or ethics committee who approved or waived your study, as well as whether or not you obtained informed written or verbal consent. If consent was waived for your study, please include this information in your statement as well.**

*** This information is provided in the Method section of the manuscript.*

6. **Please include captions for your Supporting Information files at the end of your manuscript, and update any in-text citations to match accordingly. Please see our Supporting Information guidelines for more information: http://journals.plos.org/plosone/s/supporting-information.**

*** The supporting information has been revised* ***within the manuscript to reflect journal requirements.***

**Reviewer(s)' Response to Questions:**

1. **Is the manuscript technically sound, and do the data support the conclusions?**

**Reviewer #1: Yes**

*** No action is required.*

2. **Has the statistical analysis been performed appropriately and rigorously?**

**Reviewer #1: Yes**

*** No action is required.*

3. **Have the authors made all data underlying the findings in their manuscript fully available?**

**Reviewer #1: No**

*** The URL to the data is provided in the Data Availability section of the submission portal.*

4. **Is the manuscript presented in an intelligible fashion and written in standard English?**

**Reviewer #1: Yes**

*** No action is required.*

Reviewer #1: This study examined the relationship between perceived self-stigma related to substance use disorders and engagement in substance use among non-clinical populations. The results showed that individuals with lifetime use but no substance use in the last 30-day reported higher levels of self-stigma. Perceived impact of SUD on sense of self-efficacy and self-esteem related negatively to nearly all observed factors of alcohol and marijuana use. The study suggests that increased awareness of the consequences of SUD on the sense of self has an impact on alcohol and marijuana use among young adults.

Overall, the background is well-written and provide a clear overview of the current state of knowledge regarding substance use stigma and the specific research questions addressed in the present study. The background also effectively highlights the gap in the literature regarding the role of self-stigma in the development of SUDs and the need for more research in this area. The background also provides a good introduction to the study's hypotheses and research questions, outlining the specific aims of the study and how they relate to the broader literature on substance use stigma. The use of the theory of planned behavior as a framework for understanding the relationship between self-stigma and substance use outcomes is a useful approach and shows that the study is grounded in a strong theoretical foundation. The background provides appropriate citations to support the claims made, and the use of direct quotes from previous literature adds credibility to the discussion. The authors did good job in outlining their methods, result, and discussion. However, based on the information provided, here are some recommendations for the authors:

*** We appreciate the laudatory remarks. We thank the reviewer for their insightful comments, which we believe, improved our article significantly.*

1) The authors discuss their findings within the scope of SUD; however, they only measured alcohol and marijuana. It is not scientifically sound to pinpoint two substances only and generalize it to SUD. My recommendation is to revise your title and hypothesis to indicate in within alcohol and marijuana instead of SUD.

*** Thank you for this observation. We have revised the title and various portions of the manuscript to specify alcohol and marijuana rather than substances broadly.*

2) Clarify the study design and sampling method: The authors should provide more information on how participants were recruited, the inclusion/exclusion criteria, and any potential biases in the sample.

*** As this manuscript is meant to be read as a brief report, details on design and sampling methods are kept general. However, we added some general information about the sample and also included the citation for another manuscript (and the URL per journal requirements) that provides greater detail on study design.*

3) Provide more detail on the measures used: While the authors briefly described the measures used, it still misses important information such as the operational definitions and elements of NSE, NSEF, B-MACQ; CUDIT-R, etc.

*** As this manuscript is meant to be read as a brief report, details on design and sampling methods are kept general. Further information on the measures used in this study are also available in the manuscript referenced within the method section for additional information. However, we also added some additional detail about each of the relevant measures to the present research.*

4) Discuss implications and future directions: While the authors briefly discussed the implications of their findings, they could provide a more in-depth discussion of how their results could inform future research or interventions for substance use disorders. Additionally, they could discuss any potential limitations of their study and areas for future research.

*** Thank you for these suggestions. Although this manuscript was intended to be read as a brief report, we have added additional text regarding implications, limitations, and future research.*
