# Supplementary material for: Exploring perceptions of self-stigma of substance use and current alcohol and marijuana use patterns among college students
Source: PLoS One. 2024 Apr 5;19(4):e0301535. doi: 10.1371/journal.pone.0301535 (PMC10997109; doi:10.1371/journal.pone.0301535)
Supplement: Attachment — Submitted filename: Response to Reviewers.docx [file pone.0301535.s003.docx]

**Dr. Lakshit Jain,**

**We would like to thank you for the thorough review of our manuscript and for the selection of well-qualified reviewers. We have addressed the reviewer’s concerns and summarized our changes below. By addressing the general journal requirements and reviewers’ comments, we believe our manuscript has been strengthened and will make more of a contribution to the field.**

**Journal (s)' Comments/Requirements:**

1. **Please review your reference list to ensure that it is complete and correct. If you have cited papers that have been retracted, please include the rationale for doing so in the manuscript text, or remove these references and replace them with relevant current references. Any changes to the reference list should be mentioned in the rebuttal letter that accompanies your revised manuscript. If you need to cite a retracted article, indicate the article’s retracted status in the References list and also include a citation and full reference for the retraction notice.**

*** We have reviewed our reference list and assure that is complete and correct. Minor changes were made to the formatting of the references (e.g., writing references in sentence-case). Further, no studies cited have been redacted to the best of our knowledge.*

**Additional Editor Comments:**

1. **Thank you for revising your article and making edits as recommended. Unfortunately, there are still some minor concerns that need to be addressed. Please specifically address concerns raised by reviewer 3 and 7.**

**Thanks, Lakshit**

*** We have addressed each reviewers’ concerns and summarized our changes below. By addressing the reviewer’s comments, we believe our manuscript has been strengthened and will make more of a contribution to the field.*

**Reviewer(s)' Response to Questions:**

1. **If the authors have adequately addressed your comments raised in a previous round of review and you feel that this manuscript is now acceptable for publication, you may indicate that here to bypass the “Comments to the Author” section, enter your conflict of interest statement in the “Confidential to Editor” section, and submit your "Accept" recommendation.**

**Reviewer #2: (No Response)**

**Reviewer #3: All comments have been addressed**

**Reviewer #4: All comments have been addressed**

**Reviewer #5: All comments have been addressed**

**Reviewer #6: All comments have been addressed**

**Reviewer #7: (No Response)**

*** No action is required.*

2. **Is the manuscript technically sound, and do the data support the conclusions?**

**Reviewer #2: Yes**

**Reviewer #3: Yes**

**Reviewer #4: Yes**

**Reviewer #5: Yes**

**Reviewer #6: Yes**

**Reviewer #7: Yes**

*** No action is required.*

3. **Has the statistical analysis been performed appropriately and rigorously?**

**Reviewer #2: I Don't Know**

**Reviewer #3: Yes**

**Reviewer #4: Yes**

**Reviewer #5: Yes**

**Reviewer #6: I Don't Know**

**Reviewer #7: Yes**

*** No action is required.*

4. **Have the authors made all data underlying the findings in their manuscript fully available?**

**Reviewer #2: Yes**

**Reviewer #3: Yes**

**Reviewer #4: Yes**

**Reviewer #5: Yes**

**Reviewer #6: Yes**

**Reviewer #7: Yes**

*** No action is required.*

5. **Is the manuscript presented in an intelligible fashion and written in standard English?**

**Reviewer #2: Yes**

**Reviewer #3: Yes**

**Reviewer #4: Yes**

**Reviewer #5: Yes**

**Reviewer #6: Yes**

**Reviewer #7: Yes**

*** No action is required.*

**Review Comments to the Author:**

Reviewer #2: This is a well-written manuscript. I liked reading this manuscript and believe that it is very promising. At the same time, I identified couple of issues that require the authors’ attention. It is interesting to see the correlation between pre-clinical substance use, stigma and its impact on individuals' future. Professional use of English language is at par. The manuscript is based on impressive empirical evidence and makes an original contribution but there should be some comment on possible bias like reporting bias of the study participants and also their understanding of the difference between pre-clinical substance uses and SUD itself. It will be very interesting to see the follow up study on these subject individuals regarding how many of these develops SUD.

*** We appreciate the laudatory remarks. We thank the reviewer for their insightful comments, which we believe improved our article significantly. Regarding the issues about bias in reporting, we have now added this as a limitation within the “limitations and future research” section. Specifically, we now state, “Further, the possibility of the presence of reporting bias should be of note for future research. Self-reports of stigma may have been complicated as the distinction between SUD and pre-clinical substance use was not explicitly defined to participants in this study. Similarly, the presence of SUD related stigma itself may have primed under- or over- reporting of substance use” (see p. 13).*

Reviewer #3: The authors have addressed most of the review comments except as stated below. Once the pending comment is addressed the article may be accepted for publication.

*** We appreciate the laudatory remarks. We thank the reviewer for their insightful comments, which we believe improved our article significantly.*

1) The authors discuss their findings within the scope of SUD; however, they only measured alcohol and marijuana. It is not scientifically sound to pinpoint two substances only and generalize it to SUD. My recommendation is to revise your title and hypothesis to indicate in within alcohol and marijuana instead of SUD.

The title is more clear and the hypothesis is now more sound

*** We appreciate the feedback.*

2) Clarify the study design and sampling method: The authors should provide more
information on how participants were recruited, the inclusion/exclusion criteria, and any
potential biases in the sample.

The authors have clarified this comment.

*** We appreciate the feedback.*

3) Provide more detail on the measures used: While the authors briefly described the
measures used, it still misses important information such as the operational definitions
and elements of NSE, NSEF, B-MACQ; CUDIT-R, etc.

The authors have clarified this comment.

*** We appreciate the feedback.*

4) Discuss implications and future directions: While the authors briefly discussed the
implications of their findings, they could provide a more in-depth discussion of how
their results could inform future research or interventions for substance use disorders.
Additionally, they could discuss any potential limitations of their study and areas for
future research.

The authors have clarified this comment. However, the current manuscript brings an important question. The authors themselves report how in pre problematic use population self stigma may prevent problematic use but in patients with Use Disorders they may serve as a deterrent to seek treatment. The authors may identify this as a study area where populations can be compared, or stratify their data and present the results of stigma in groups stratified on the basis of amount/frequency of use or substance of use (alcohol or cannabis).

This would make this paper more methodologically rigorous and increase the scientific impact of this paper. I look forward to reading the revised manuscript.

*** The reviewer makes an astute point. Comparing perceived self-stigma scores across amount/frequency or substance of use could add to this paper; however, this would be complicated by the fact that our perceived self-stigma variable is based on “substance use disorder” broadly and making such comparisons may not be as methodologically sound. Nonetheless, this is an important point, and we are actively collecting data on a follow-up study where we actually break down “perceived self-stigma” to focus on alcohol and cannabis specific use disorders. Thus, within those new data we can better stratify and determine whether frequency/amount of use of that drug actually impacts perceived stigma of that specific drug use disorder.*

*Regarding the comparisons between pre-clinical and clinical samples, we have further emphasized in the future directions section that more work comparing across pre-clinical and clinical samples is needed to determine the overall impact self-stigma (and stigma more broadly) has on substance use patterns. Specifically, we now state, “Future research may also try to explicitly identify distinct conditional associations of perceived self-stigma with substance use practices between clinical and non-clinical populations, as well as strata within non-clinical populations (e.g., people who use heavily daily, people who use lightly monthly)” (see p. 14).*

Reviewer #4: Thank you for the opportunity to review this brief report that examines the relationship between self stigma related to alcohol and MJ use and engagement in use in college students. The manuscript is well written, and succinct and the background section explains the rationale for the study well. The findings are clearly communicated. The authors seem to have addressed the concerns of the previous reviewer well making significant changes especially to the discussion section which was well written, especially the limitations. The main finding of the study that shows that self stigma could potentially play a protective role against use is valuable and an important contribution to the literature. I don’t have any other major edits/suggestions. Great work.

*** We appreciate the laudatory remarks. We thank the reviewer for their insightful comments, which we believe improved our article significantly.*

Reviewer #5: The research paper details the relationship between the self-stigma of marijuana and alcohol use and engagement in these substances among college students. It Indicates that individuals with lifetime use of marijuana and alcohol, but no recent use reported higher self-stigma. Negative self-esteem and self-efficacy were found to relate negatively to nearly all observed factors of alcohol and marijuana use.

Feedback:
The assertion positing a link between self-stigma and involvement in substance use is substantiated by robust research findings, which reveal that individuals who have a history of substance use but no recent use exhibits elevated levels of self-stigma. This solid empirical basis lends credence to the argument, rendering it valid. Likewise, the claim proposing a negative relationship between negative self-esteem and self-efficacy with alcohol and marijuana use is bolstered by the study's outcomes. The argument finds strong empirical support in the research data, further establishing its validity. Moreover, the argument asserting that an enhanced awareness of the impact of substance use disorders on an individual's sense of self can influence alcohol and marijuana use is well-founded in the study's results. This logical reasoning harmonizes effectively with the study's objectives, thus underscoring its validity.

The authors have diligently responded to the concerns raised in the previous review and have effectively rectified the identified issues. Their commitment to addressing these concerns is evident in the revised manuscript. They have demonstrated a high level of responsiveness and professionalism in dealing with the feedback, which reflects positively on the overall quality of the research. The corrections made by the authors have notably improved the clarity and coherence of the manuscript. The revised version now presents a more refined and accurate portrayal of their research findings, strengthening the overall credibility and impact of their work. The authors' attention to detail and their willingness to make the necessary revisions exemplify their commitment to producing a high-quality academic contribution. This constructive engagement with feedback not only benefits the authors by enhancing the quality of their work but also contributes to the scholarly community by ensuring that accurate and reliable research is disseminated. It is evident that the authors' dedication to addressing the concerns raised in the previous review will enhance the value and relevance of their research within the academic field.

*** We appreciate the laudatory remarks. We thank the reviewer for their insightful comments, which we believe improved our article significantly.*

Reviewer #6: (No Response)

*** We thank the reviewer for their review.*

Reviewer #7: Thanks for giving me the opportunity to review this article. The article is interesting as it explores the protective effect of self stigma on alcohol and marijuana use. This is a rare concept that has not been explored much so far. The article is very unique in that aspect. The sample size is excellent. Also the author has been very candid in acknowledging the limitation of the study that majority of participants were females. It would be interesting to see the results of the study if all genders were included( Including LGBT etc)

*** We thank the reviewer for their insightful comments, which we believe improved our article significantly. We also agree that examining these relationships among diverse populations is a needed area of research in our future work and are committed to supporting expansion of research into this area of intersectional identities.*

1. One limitation of the study is the study included college students. There are a lot of students that drop out of high school due to substance use problems and it would be great to know how their self stigma compares to someone who has made it to college.

SOLUTION: Please include in the limitation that perhaps including the college going students have skewed the results of the study and the results may not be extrapolated to the entire population.

*** The reviewer makes an astute point. Regarding the issues about high school students and that our sample does not represent all college students, we have now added this as a limitation within the “limitations and future research” section. Specifically, we now state, “It is also important to highlight that this research was intentionally focused on a non-clinical college population and thus cannot be extrapolated to broader populations” (see p. 13). We also state, “Future studies can examine these relationships more deeply to establish causality, how self-stigma can influence substance use and related behavioral or functional outcomes longitudinally (e.g., education completion), and identify the specific factors within self-stigma that may be protective so that clinicians and public health experts alike can leverage those factors specifically in campaigns and legislature averting development of SUD” (see p.14).*
