# Supplementary material for: Exploring perceptions of self-stigma of substance use and current alcohol and marijuana use patterns among college students
Source: PLoS One. 2024 Apr 5;19(4):e0301535. doi: 10.1371/journal.pone.0301535 (PMC10997109; doi:10.1371/journal.pone.0301535)
Supplement: Attachment — Submitted filename: Response to Reviewers.docx [file pone.0301535.s004.docx]

**Dr. Lakshit Jain,**

**We would like to thank you again for the thorough review of our manuscript and for the selection of well-qualified reviewers. We have addressed the reviewer’s concerns and summarized our changes below. By addressing the general journal requirements and reviewers’ comments, we believe our manuscript has been strengthened and will make more of a contribution to the field.**

**Editor Comments:**

1. **Three reviewers have recommend to accept this article, but reviewer 8 is seeking this article for the first time, they have raised multiple concerns that should be resolved prior to accepting this article**

*** We have addressed each reviewer 8’s concerns and summarized our changes below. By addressing the reviewer’s comments, we believe our manuscript has been strengthened and will make more of a contribution to the field.*

**Reviewer(s)' Response to Questions:**

1. **If the authors have adequately addressed your comments raised in a previous round of review and you feel that this manuscript is now acceptable for publication, you may indicate that here to bypass the “Comments to the Author” section, enter your conflict of interest statement in the “Confidential to Editor” section, and submit your "Accept" recommendation.**

**Reviewer #2: All comments have been addressed**

**Reviewer #3: All comments have been addressed**

**Reviewer #5: All comments have been addressed**

**Reviewer #8: (No Response)**

*** No action is required.*

2. **Is the manuscript technically sound, and do the data support the conclusions?**

**Reviewer #2: Yes**

**Reviewer #3: Yes**

**Reviewer #5: Yes**

**Reviewer #8: Partly**

*** No action is required.*

3. **Has the statistical analysis been performed appropriately and rigorously?**

**Reviewer #2: Yes**

**Reviewer #3: Yes**

**Reviewer #5: Yes**

**Reviewer #8: I Don't Know**

*** No action is required.*

4. **Have the authors made all data underlying the findings in their manuscript fully available?**

**Reviewer #2: Yes**

**Reviewer #3: Yes**

**Reviewer #5: Yes**

**Reviewer #8: Yes**

*** No action is required.*

5. **Is the manuscript presented in an intelligible fashion and written in standard English?**

**Reviewer #2: Yes**

**Reviewer #3: Yes**

**Reviewer #5: Yes**

**Reviewer #8: Yes**

*** No action is required.*

**Review Comments to the Author:**

Reviewer #2: (No Response)

*** We appreciate the laudatory remarks. We thank the reviewer for their insightful comments, which we believe improved our article significantly.*

Reviewer #3: The authors have sufficiently and succinctly made appropriate changes to the manuscript to make it more reader friendly. The paper presents an interesting approach to alcohol and cannabis use disorder propagation and at this stage would benefit from acceptance in the journal. Thank you for the opportunity to review this paper.

*** We appreciate the laudatory remarks. We thank the reviewer for their insightful comments, which we believe improved our article significantly.*

Reviewer #5: The paper has improved after the revisions. The authors' meticulous efforts have substantially strengthened the manuscript, aligning it more closely with the standards of PLOS ONE.

*** We appreciate the laudatory remarks. We thank the reviewer for their insightful comments, which we believe improved our article significantly.*

Reviewer #8: This manuscript presents information on college students’ perception of self- stigma associated with developing a substance use disorder (SUD) in the context of alcohol and marijuana use. It is well written and below are my suggestions on how the manuscript can be improved.

***We thank the reviewer for their insightful comments, which we believe improved our article significantly.*

Abstract
Generally, the abstract would benefit from a structure; introduction of different sections will improve focus and clarity

***We have updated the abstract to make it more structured. Specifically, we have now added qualifiers for each section: background, aims, method, results, and conclusions. We hope these changes will improve the focus and clarity of our abstract section of the paper.*

Line 32
There is no mention of the instruments used to collect the data.

***Given word limitations by the journal for abstracts, we did not want to write out every measure that was used to assess study constructs as it would push us past the word limit for the abstract based on the journal requirements.*

Line 34 – 40
The results in this section require the support of the numbers that tell the story. Presentation could benefit by stating the actual figures obtained in the statistical analysis, including the p values while mentioning the significance levels.
For instance, were the higher levels of self- stigma mentioned in Line 37- 38 statistically significant? What were the actual figures?

***While we normally agree with this point, there are numerous correlations that would need to be presented for each outcome. Given the journal requirements regarding word limit for an abstract, including all of these statistics would push us past that limit. Thus, why they are omitted, and we defer to qualitative explanations of study results.*

Line 44- 46
“Rather, we interpret these findings to indicate that increased awareness of the consequences of substance use disorder on the sense of self has an impact on alcohol and marijuana use among young adults”. When one has the objectives of the study in mind, it appears the different patterns of alcohol and marijuana use are actually the independent variables with self- stigma, and not awareness, as the dependent variable. This may require clarification.

The conclusion of “increased awareness” cannot emanate from the findings of this particular study; there was no measure of awareness of the consequences of SUD. What was measured here was the self- stigma. Additionally, the increased awareness in this context sounds more like insight into the consequences of SUD.

***We appreciate this comment and agree with the reviewer. We have rephrased this sentence such that it now reads, “Rather, we interpret these findings to indicate that negative perceptions of substance use disorder on the sense of self may be associated with distinct alcohol and marijuana use behaviors among young adults”.*

Introduction
The use of alcohol use disorder (AUD), marijuana use, and SUD interchangeably in this section may be problematic. For instance, Line 70- 71; “problematic marijuana use may culminate in a SUD”; why not a cannabis use disorder (CUD)?

***We have carefully updated the introduction to be more focused on when we are describing AUD, CUD, and SUD.*

Measures
Line 122-126
The statement …we replaced the term “mental illness” with “substance use disorder” throughout the measure…is worrying. Were the psychometric properties of the new instrument, created by replacing terminology, ascertained or was it an assumption that they were acceptable? Was there a definition of the term SUD that was provided to all the participants, or were they expected to respond using their own individual definitions and interpretation of what SUD meant- not a standardized one? Was it actually meant to be AUD, CUD or the actual DSM-5 TR SUD? Is there a possibility that the participants understood it to mean opioids, cocaine, nicotine, inhalants, hallucinogen and other substance use disorders? Was this how it was intended to be understood? Did SUD mean severity? Was a pilot on the instrument ever done after changing the terminology, to establish reliability and validity? So, do we actually know what was measured by this novel measure/ instrument and what the content of the results we are discussing here are?

***The reviewer raises several good points. Regarding the validity of this change to the prior measure, we wrote in the paragraph describing this measure that we conducted EFA analyses to examine its factor structure. We also provided more information to these analyses in S1 Appendix and we refer the reviewer to that section for more clarity of the reliability/validity of the measure.*

*Regarding the conceptual aspects, we purposely wanted to get people’s perceptions of SUD broadly and not tied to one drug. To that end, we purposely did not define SUD for participants as this definition varies across drug types and even cultures. Nonetheless, there is a possibility that this created issues in how people responded if they were focusing on one particular drug (e.g. opioids). We touch upon each of these issues in the limitations section and suggest ways to improve upon our study in future research (see pgs. 12-14).*

If this was the thrust of the study, why not use ‘perception of self- stigma associated with developing a SUD in the context of substance use’, instead of alcohol or cannabis use? Alternatively, use ‘perception of self- stigma associated with developing an AUD/ CUD in the context of their use’. This would enable us to confidently compare “apples” with “apples” and not “oranges”.

***Our original title was “Exploring Perceptions of Self-Stigma of Substance Use and Current Substance Use Patterns among College Students”. However, a different reviewer felt that it was too broad considering we only assessed alcohol and cannabis variables in correlating with SUD stigma perception. Thus, why the title is currently: “Exploring perceptions of self-stigma of substance use and current alcohol and marijuana use patterns among college students”.*

*Nonetheless, the reviewer is correct that future work should be tailored to specific perceptions of use disorder for a specific drug. Further, examining stigma perceptions of that particular drug (e.g., AUD) and how those perceptions associate with that drug use (e.g., alcohol use patterns) is important.*

Results

From the presentation of the results, it is clear that the participants’ socio-demographic characteristics were not considered, even with the obvious gender imbalance. Despite the fact that this was not part of the objectives, it would have been nice to know their residence, living arrangements, finances, marital status, educational level of study, age, and other characteristics that could be factors associated with the outcome.

***The reviewer is correct in that our focus was not to examine sociodemographic predictors of perceptions of stigma related to SUD. We do describe some demographic information (age, gender, race/ethnicity) about the sample within the “participants and procedure” section. We don’t have information on marital status, residence, and finances which is why this information was not reported.*

Line 208- 209

Table 1- I suggest that the “significant differences” column is given a figure/ value/ number, to denote and support the significance level. The explanation given in the table is a repetition (see text in Line 203- 207) and should be removed.

***Given that we already present the statistics about the omnibus ANOVA results in text, we did not feel it was appropriate in Table 1. Furthermore, we believe it is easier for readers who are not savvy in statistics to be able to digest and interpret the significant statistical results in a clearer manner. Further, we believe by reporting in the note section how statistical significance was determined, it can place in context our results for readers.*

Line 222- 223

Table 2- Were there scatter plots done to establish linearity? Does 1,2, 3…. columns represent the corresponding 1,2, 3…. rows in the bi-variate correlations? If so, it does not reflect the expected quality that a variable is always perfectly correlated with itself (r=1). May require clarification.

***As mentioned in the note section of Table 2: Cronbach’s alpha for each measure is underlined and shown on the diagonal. We feel like this is more important information than just showing that each variable correlates perfectly with itself.*

Discussion

This is largely determined by the findings; I have highlighted the concern in the findings. However, it was easy to notice that the limitations and future research section (Line 260- 309) was twice as long as the discussion (238- 258). Separating the limitations from the future research, so that they are two distinct sections could improve clarity. Future research begins from Line 270; although there is a small section, Line 277- 281 which is mainly focused on limitations

***We appreciate this point. However, we believe that by raising the limitations and then directly mentioning how these limitations can be improved upon in future research is easier for readers to digest. In other words, we did not want to list many limitations and then jump into a new section focused on future research that jumps around as opposed to focusing on how that future direction can alleviate current limitations of the present study.*
